# Effect of pH on the Aggregation of α-syn12 Dimer in Explicit Water by Replica-Exchange Molecular Dynamics Simulation

**DOI:** 10.3390/ijms160714291

**Published:** 2015-06-24

**Authors:** Zanxia Cao, Xiumei Zhang, Lei Liu, Liling Zhao, Haiyan Li, Jihua Wang

**Affiliations:** 1 Shandong Provincial Key Laboratory of Functional Macromolecular Biophysics, Dezhou 253023, China; E-Mails: zhangxiumei@dzu.edu.cn (X.Z.); zhaoll@sina.com (L.Z.); tianwaifeixian78@163.com (H.L.); jhw25336@126.com (J.W.); 2 College of Physics and Electronic information, Dezhou University, Dezhou 253023, China; 3 College of Computer Science and Technology, Dezhou University, Dezhou 253023, China; E-Mail: leiliusid@gmail.com

**Keywords:** α-synuclein, different pH, dimerization, structural character, molecular dynamics simulation

## Abstract

The dimeric structure of the N-terminal 12 residues drives the interaction of α-synuclein protein with membranes. Moreover, experimental studies indicated that the aggregation of α-synuclein is faster at low pH than neutral pH. Nevertheless, the effects of different pH on the structural characteristics of the α-syn12 dimer remain poorly understood. We performed 500 ns temperature replica exchange molecular dynamics (T-REMD) simulations of two α-syn12 peptides in explicit solvent. The free energy surfaces contain ten highly populated regions at physiological pH, while there are only three highly populated regions contained at acidic pH. The anti-parallel β-sheet conformations were found as the lowest free energy state. Additionally, these states are nearly flat with a very small barrier which indicates that these states can easily transit between themselves. The dimer undergoes a disorder to order transition from physiological pH to acidic pH and the α-syn12 dimer at acidic pH involves a faster dimerization process. Further, the Lys6–Asp2 contact may prevent the dimerization.

## 1. Introduction

In Parkinson’s disease, the main component of the amyloid deposits found in Lewy bodies has been identified as α-synuclein protein, an intrinsically disorder protein with 140 residue acids [1,2]. The aggregation of α-synuclein protein [3,4,5] and membrane binding [6] are two critical factors which led to Parkinson’s disease. The dimerization of α-synuclein is a key step in the aggregation progress [7], and the dimeric structures of α-synuclein bind preferentially with the lipid membrane in comparison to the monomeric protein [8]. Thus, exploration of the dimeric structures of α-synuclein are of significant interest.

The N-terminal region (residues 1–60) of the α-synuclein protein, is involved in the dimerization and plays a key role in the formation of α-synuclein assemblies [9]. Specifically, the 11 residues at the N-terminal are crucial for monomers’ and oligomers’ interactions with membranes [10]. Moreover, experimental studies showed that α-synuclein aggregates faster at low pH than neutral pH [11]. However, the effects of different pH on the structural characteristics of the α-syn12 dimer still remain poorly understood. Hence, it may provide deep comprehension of the aggregation and the membrane binding if we explore the dimeric structures and the dynamic conformation of the α-syn12 dimer (1–12 residues of α-synuclein protein) at different pH.

Molecular dynamics simulation is a valid method for investigating the dimer structural character of amyloid peptide, such as polyglutamine peptide, amyloid-β protein (Aβ), human Amylin, and α-synuclein *etc.* Chiang *et al.* [12] revealed how the polyglutamine dimers form the β-sheet and found that the dimers tends to form the anti-parallel β-sheet conformations rather than the parallel β-sheet by using all-atom replica exchange molecular dynamics (REMD) simulations. Since Alzheimer’s disease is associated with the Aβ protein, the dimerization of this protein or fragment receives more attention. Shea *et al.* [13] reviewed the molecular dynamic simulations of the Aβ protein. Barz *et al.* [14] compared the structural character of Aβ40 and Aβ42 monomers and dimmers. The free energy surface of the Aβ42 dimer indicated a larger conformational variability in comparison to the Aβ40 dimer. Zhu *et al.* [15] and Chong and Ham [16] investigated the mechanism of dimerization of the full-length Aβ42 peptide by using classical molecular dynamics simulation. The simulations found the three most stable dimers and indicated that the hydrophobic regions play critical roles in the dimerization process. A number of investigations of Aβ fragments such as Aβ21–30, Aβ16–22 and Aβ25–35), by Smith *et al.* [17], Cruz *et al.* [18], and Nguyen *et al.* [19], respectively, and others were reported, which further characterized the structure character of the dimer. The results showed that Aβ21–30 form both anti-parallel and parallel dimer spontaneously, and the anti-parallel state is more stable than parallel. For Aβ16–22 [18] and Aβ25–35 [19], the dimers form a diverse ensemble including anti-parallel and parallel β-sheets. Qi *et al.* [20] revealed the conformational distribution and the conformational transition of the human islet amyloid polypeptide (HIAPP) (11–25) dimer from α-helix to β-sheet by using all-atom REMD simulations. The β-sheet structures are mostly anti-parallel, and the hydrophobic interactions play an important role in the dimerization process. Guo *et al.* [21] revealed the structures of the HIAPP (22–28) dimer and oligomers. The hydrophobic interactions and backbone hydrogen bond interaction play an important role in the stabilization. Eugene *et al.* [22] characterized the structural change of the α-synuclein 61–95 region from the monomer to the dimer and the trimer by using coarse-grained REMD simulations. The dimer and the trimer form more β-sheet structure and display a strong polymorphism.

In this article, we are interested in characterizing the dimer states of the α-syn12 at acidic pH and physiological pH. Our early studies [23,24,25] indicated that the α-syn12 monomer peptide adopts a β-hairpin as the most clustered structure at different pH levels. At physiological pH, the β-hairpin was with turn 9-6 and four hydrogen bonds (HB9–6, HB6–9, HB11–4 and HB4–11), and two hydrophobic residues (Phe4 and Ala11) involved. At acidic pH, the β-hairpin was with turn 8-5 and five hydrogen bonds (HB8–5, HB5–8, HB10–3, HB3–10 and HB12–1), and four hydrophobic residues (Met1, Val3, Met5 and Leu8) involved.

Here we use T-REMD simulations with explicit solvent to study the structural character of α-syn12 dimer at acidic pH and physiological pH. The inter-molecular states of α-syn12 dimer are determined by constructing the free energy surface based on a series of reaction coordinates. These free energy surfaces are further used to pick-up the highly populated states. The results reported in this study will provide a deeper understanding of the dimerization process.

## 2. Results and Discussion

### 2.1. Effect of pH on the Inter-Molecular States of (α-syn12)_2_

We characterized the inter-molecular states of (α-syn12)_2_ using the new method Nguyen *et al.* [26] proposed and other traditional methods.

#### 2.1.1. Free Energy Surface Obtained from PCA of Inter-Chain Side-Chain Inverse Distances

**Figure 1:**
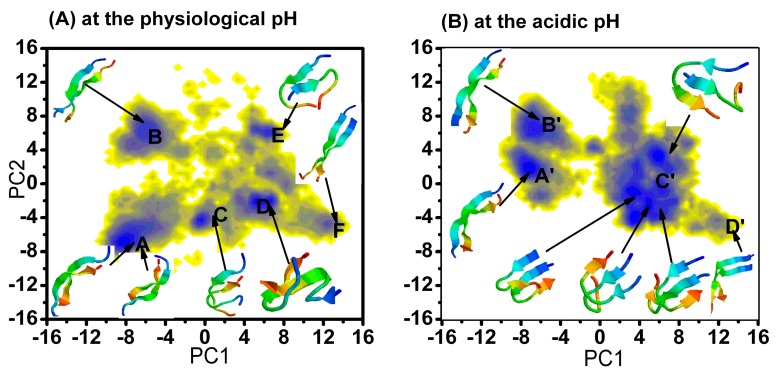
Free energy surface (in kJ/mol) of the (α-syn12)_2_ dimer (**A**) at the physiological pH and (**B**) at the acidic pH projected on the first two principal components PC1 and PC2 obtained from PCA of inter-chain side-chain inverse distances. The blue indicates the N-terminal.

We used the method developed by Nguyen *et al.* [26], which was also used to describe the dimer of Aβ40 and Aβ42 [27,28]. For the (α-syn12)_2_ dimer, there are 144 distances between the inter-chain centers of mass of the side-chains. These distances completely specify the inter-molecular structures of a conformation of the dimer. We then applied the PCA of the inverse distances on the combined trajectories at two different pH values for the last 300 ns. The free energy surface (Figure 1) was constructed using the first two principle components (PC1 and PC2) as the reaction coordinates. The first two components that contribute to the total fluctuation are 26% and 15%. The relative depths of the free energy minima of α-syn12 dimer at different simulation time are given in Table 1 and Table 2. The corresponding representative structures are shown in Figure 1. The representative structures are selected by using a linkage clustering method based on the root-mean-square deviations in the positions of Cα atoms (RMSDCα); the cut-off is set as 0.2 nm.

For the last 300 ns of the simulation at physiology pH and 300 K, there were six highly populated regions on the PCA map (cited as A–F in Figure 1), the corresponding free energy were −15.6, −13.1, −12.0, −15.5, −13.8 and −11.5 kJ/mol, the relative depths were 0.0, 2.5, 3.6, 0.1, 1.8 and 4.1 kJ/mol, respectively. The main differences among these six representative structures were the inter-side chain interactions and inter-backbone hydrogen bonds. State A is the global minimum state in this free energy surface. This state is an anti-parallel inter-peptide β-sheet, the corresponding representative structures have eight inter-molecular hydrogen bonds (HB). States B, C and E are also anti-parallel inter-peptide β-sheets with different inter-backbone hydrogen bonds. State F is parallel inter-peptide β-sheets. The results indicated that the dimer tends to form various β-sheet conformations, including the anti-parallel and the parallel. The anti- parallel and the parallel structures are independently located at the local minimums. Additionally, the six highly populated regions are nearly flat with a very small barrier indicating that these states are widely varied in structure and can easily transition between themselves.

**Table 1:** The relative depths of the free energy minima of the α-syn12 dimer at the physiological pH.

The Different Regions	The Relative Depths (kJ mol^−1^) of the Minima at Physiology pH
	201–500 ns
A	0
B	2.5
C	3.6
D	0.1
E	1.8
F	4.1

**Table 2:** The relative depths of the free energy minima of the α-syn12 dimer at the acidic pH.

The Different Regions	The Relative Depths (kJ mol^−1^) of the Minima at Acidic pH
	201–500 ns
A'	0
B'	1.1
C'	−0.6
D'	6.1

For the last 300 ns of the simulation at acidic pH and 300 K, there were four highly populated regions on the PCA map (cited as A'–D' in Figure 1), the corresponding free energy values were −14.2, −13.1, −14.8 and −8.1 kJ/mol; and the relative depths were 0.0, 1.1, −0.6 and 6.1 kJ/mol, respectively. States A' and C' are the global minimum state in this free energy surface. State A' and B' are anti-parallel inter-peptide β-sheet with different inter-backbone hydrogen; the corresponding representative structures have eight inter-molecular hydrogen bonds (HB). State D' is a parallel inter-peptide β-sheet with very high free energy. Thus, it is reasonable to conclude that the free energy surface at acidic pH only contains three highly populated regions.

The only difference for the simulation system at physiological pH and acidic pH is the net charge of the second residue (Asp2). That net charge per residue modulates conformational ensembles of intrinsically disordered proteins (IDPs) was demonstrated by Mao *et al.* [29]. The above free energy surfaces indicate the dimer undergoes a disorder to order transition by the change of the net charge of Asp2.

#### 2.1.2. Free Energy Surface Based on the Other Representative Reaction Coordinates

**Figure 2:**
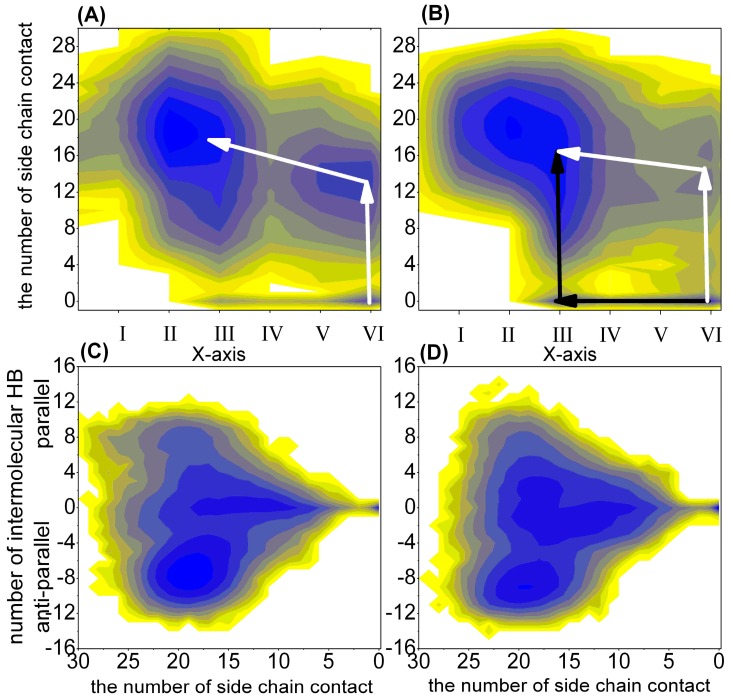
The free energy surface (in kJ/mol) of the (α-syn12)_2_ dimer projected on other representative reaction coordinates. (**A**) is the free energy surface based on the *X*-axis and the number of side chain contact at physiological pH; (**B**) is the free energy surface based on the *X*-axis and the number of side chain contact at acidic pH; (**C**) is the free energy surface based on the number of side chain contact and the number of intermolecular hydrogen bond at physiological pH; and (**D**) is the free energy surface based on the number of side chain contact and the number of intermolecular hydrogen bond at acidic pH The *X*-axis represents the existence of the following: (VI), NUMintraHB > 0, Lhelix > 0, Lsheet = 0; (V), NUMintraHB > 0, Lhelix > 0, Lsheet > 0; (IV), NUMintraHB > 0, Lhelix = 0, Lsheet = 0; (III), NUMintraHB > 0, Lhelix = 0, Lsheet > 0; (II), NUMintraHB = 0, Lhelix = 0, Lsheet > 0; and (I), NUMintraHB = 0, Lhelix = 0, Lsheet = 0.

The above analysis suggested that the main differences among these ten representative states were the inter-side chain interactions and inter-backbone hydrogen bonds. For the purpose of understanding the secondary structure and interaction changes, we also chose the length of helix (Lhelix), the length of β-sheet (Lsheet), the number of intra-backbone hydrogen bonds (NUMintraHB), the number of inter-sidechain contacts (NUMcon) and the number of inter-backbone hydrogen bonds (NUMinterHB) as the reaction coordinates to construct the free energy surfaces (FESs). To distinguish the parallel β-sheet from the anti-parallel β-sheet, we calculated the intra-peptide end-to-end vectors and cosine of the angle between these two vectors. The last number of inter-backbone hydrogen bonds in Figure 2 is the number multiplied by the cosine of the angle. The respective probabilities for α-syn12 dimer fall within different regions at different pH and the simulation time are described in Table 3 and Table 4. For the last 300 ns of the simulation at 300 K, the dimer loses their helix conformation and adopts a β-sheet conformation. However, for the first 200 ns, the dimer at physiological pH displays a more significantly increased α-helix than the dimer at acidic pH, indicating that it is faster from α to β transition at acidic pH than at physiological pH. Furthermore, for the last 400 ns, the NUMcon are always larger than zero; for the first 100 ns, the NUMcon at physiological pH are always larger than zero at states I–V, and the probability for α-syn12 dimer at state III and NUMcon equal to zero (the dimer only form the intra β-sheet and do not form any inter-sidechain contacts) at acidic pH is 0.05. Free energy surface based on the representative reaction coordinates are shown in Figure 2. The possible transformation pathways are also noted using the arrows. The α-syn12 dimer has different transformation pathways at the different pH values. There is only one transformation pathway (the white line in Figure 2) at physiological pH; along the pathway the α-syn12 dimer forms the inter-sidechain contacts firstly, and then the β-sheet develops. However, there are two transformation pathways at acidic pH, one of the two pathways is the same as the pathway at physiological pH, and another pathway (the black line in Figure 2) has the follow characteristics, the α-syn12 dimer forms the intra-molecular β-sheet firstly, and then it forms the inter-side chain contacts. The characterization and classification of the conformations of the dimer remain difficult. For example, States A, B, C and E in Figure 1 are anti-parallel inter-peptide β-sheets with different inter-backbone hydrogen bonds. Figure 2 reveals that these states cannot be distinguished from each other. The respective probabilities for the α-syn12 dimer falling within different inter-backbone hydrogen bonds at the different pH values and simulation times are described in Table 5. The simulation at physiological pH tended to sample the full, extended anti-parallel (states A and B) and parallel β-sheet (state F) more than simulation at acidic pH. However, the simulation at acidic pH tended to sample structures not only to form the intra β-sheet but also the inter β-sheet (state C') more than simulation at physiological pH.

**Table 3:** The respective probabilities for the α-syn12 dimer fall within different regions at the different pH values and simulation times. The results at the acidic pH are shown in the brackets.

The Different Regions	1–100 ns	101–200 ns	201–300 ns	301–400 ns	401–500 ns
NUMintraHB > 0, Lhelix > 0, Lsheet = 0; Cited as VI	0.23 (0.11)	0.04 (0)	0 (0)	0 (0)	0 (0)
NUMintraHB > 0, Lhelix > 0, Lsheet > 0; Cited as V	0.17 (0.04)	0.04 (0)	0 (0)	0 (0)	0 (0)
NUMintraHB > 0, Lhelix = 0, Lsheet = 0; Cited as IV	0.04 (0.04)	0.01 (0.01)	0 (0.03)	0 (0)	0 (0.01)
NUMintraHB > 0, Lhelix = 0, Lsheet > 0; Cited as III	0.48 (0.69)	0.37 (0.54)	0.32 (0.39)	0.23 (0.41)	0.16 (0.41)
NUMintraHB = 0, Lhelix > 0, Lsheet = 0	0 (0)	0 (0)	0 (0)	0 (0)	0 (0)
NUMintraHB = 0, Lhelix > 0, Lsheet > 0	0 (0)	0 (0)	0 (0)	0 (0)	0 (0)
NUMintraHB = 0, Lhelix = 0, Lsheet > 0; Cited as II	0.07 (0.10)	0.53 (0.40)	0.67 (0.48)	0.75 (0.49)	0.82 (0.49)
NUMintraHB = 0, Lhelix = 0, Lsheet = 0; Cited as I	0 (0.02)	0 (0.04)	0 (0.09)	0 (0.09)	0.01 (0.08)

**Table 4:** The respective probabilities for the α-syn12 dimer fall within the different regions (from VI to I) at the different pH and simulation time. The results at acidic pH are shown in the brackets.

The Different Regions	1–100 ns	101–200 ns	201–300 ns	301–400 ns	401–500 ns
VI and NUM_con_ > 0	0.20 (0.06)	0.04 (0)	0 (0)	0 (0)	0 (0)
VI and NUM_con_ = 0	0.04 (0.05)	0 (0)	0 (0)	0 (0)	0 (0)
V and NUM_con_ > 0	0.17 (0.03)	0.04 (0)	0 (0)	0 (0)	0 (0)
V and NUM_con_ = 0	0 (0.01)	0 (0)	0 (0)	0 (0)	0 (0)
IV and NUM_con_ > 0	0.04 (0.04)	0.01 (0.01)	0 (0.03)	0 (0)	0 (0.01)
IV and NUM_con_ = 0	0 (0)	0 (0)	0 (0)	0 (0)	0 (0)
III and NUM_con_ > 0	0.48 (0.64)	0.37 (0.54)	0.32 (0.39)	0.23 (0.41)	0.16 (0.41)
III and NUM_con_ = 0	0 (0.05)	0 (0)	0 (0)	0 (0)	0 (0)
II and NUM_con_ > 0	0.07 (0.10)	0.53 (0.40)	0.67 (0.48)	0.75 (0.49)	0.82 (0.49)
II and NUM_con_ = 0	0 (0)	0 (0)	0 (0)	0 (0)	0 (0)
I and NUM_con_ > 0	0 (0.02)	0 (0.04)	0 (0.09)	0 (0.09)	0.01 (0.08)
I and NUM_con_ = 0	0 (0)	0 (0)	0 (0)	0 (0)	0 (0)

**Table 5:** The respective probabilities for the α-syn12 dimer fall within different inter-backbone hydrogen bonds at the different pH and simulation time. The results at the acidic pH are shown in the brackets.

The Different Regions	1–100 ns	101–200 ns	201–300 ns	301–400 ns	401–500 ns
NUMinterHB = 0	0.18 (0.14)	0 (0)	0 (0)	0 (0)	0 (0)
NUMinterHB > 0, 0° ≤ θ ≤ 50°	0.28 (0.03)	0.15 (0)	0.10 (0)	0.10 (0)	0.07 (0)
NUMinterHB > 0, 50° ≤ θ ≤ 90°	0.27 (0.24)	0.14 (0.22)	0.17 (0.25)	0.10 (0.29)	0.09 (0.20)
NUMinterHB > 0, 90° < θ < 130°	0.15 (0.23)	0.18 (0.20)	0.13 (0.21)	0.18 (0.22)	0.12 (0.25)
NUMinterHB > 0, 130° ≤ θ ≤ 180°	0.12 (0.23)	0.53 (0.42)	0.59 (0.41)	0.62 (0.38)	0.72 (0.45)

#### 2.1.3. Free Energy Surfaces for Different States

**Figure 3:**
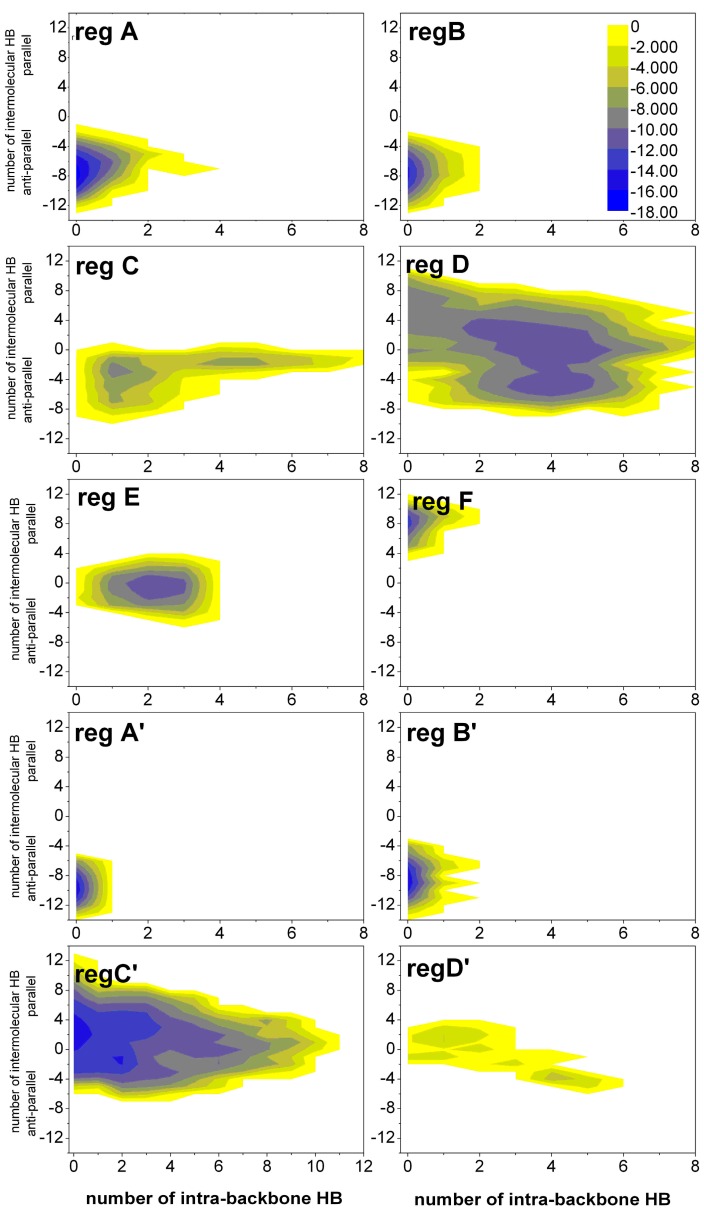
The free energy surfaces (in kJ/mol) for A–F regions of the (α-syn12)_2_ dimer at the physiological pH and A'–D' regions at the acidic pH projected on the number of intra-backbone hydrogen bonds and the number of inter-backbone hydrogen bonds.

The simulations at different pH tended to sample different states. It is difficult to discriminate these states. We select the number of intra-backbone hydrogen bonds and the number of inter-backbone hydrogen bonds as the reaction coordinates to construct the free energy surfaces (FESs) for A–F and A'–D' regions (see Figure 3). States A, B, A' and B' only have anti-parallel inter-molecular hydrogen bonds. States F only have parallel inter-molecular hydrogen bonds. However, States C, D, E, C' and D' have inter-molecular hydrogen bonds and intra-molecular hydrogen bonds.

Computation of the intra-peptide HB contact probability maps and inter-peptide HB contact probability maps (details in Supplementary Information).

#### 2.1.4. Possible Transformation Pathways

For the α-syn12 peptide monomer, our early study [23] indicated turn-directed α-helix to β-sheet conformational transitions at different pH values. For the α-syn12 peptide dimer, the anti-parallel β-sheet conformation was found as the lowest free energy state. The intra-peptide HB contact probability maps (Figures S3 and S4) and inter-peptide HB contact probability maps (Figures S5 and S6) for the first 200 ns are shown in Supplementary Information.

**Figure 4:**
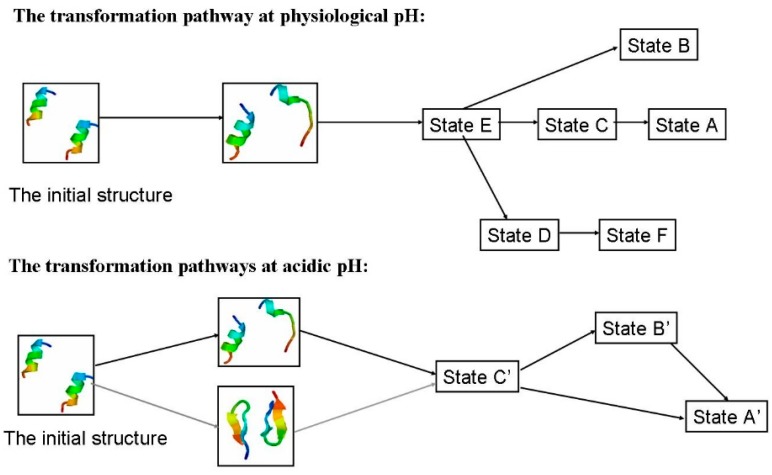
The possible transformation pathways.

Figure 4 shows the transformation pathways between different states corresponding to Figure 1 and the transition states corresponding to Figure 2. T-REMD simulations can overcome the free-energy barriers and one state can move to neighboring states. The intra-peptide HB contact probability maps (Figure S1) and inter-peptide HB contact probability maps (Figure S2) are also used to build transformation pathways. State G is composed largely of intra-peptide Lys6–Asp2 and Leu8–Met5 contacts, which can transform to states H, E, I directly and through states E and F can transform to state B and D. Although states E and H are neighbors, it is difficult to transform from state E to H or from state H to E.

### 2.2. Effect of pH on the Intra-Molecular States of (α-syn12)_2_

We analyzed the residue specific secondary structure propensity (the results are shown in Figure S7 (at physiological pH) and Figure S8 (at acidic pH).), backbone dihedral angle distribution (Figure S9) and free energy surface obtained from dPCA (see Figure S10), which specifies the intra-molecular structures of the dimer. See Supplementary Information for details.

For α-syn12 dimer at physiological pH, the helix population vanishes and a high population (larger than 60%) of β-strand is observed for residues F4-A11. A more significantly increased β-content is observed in the dimer than the monomer at residues F4-A11, especially at residues G6-L7. For α-syn12 dimer at acidic pH, the population of the helix vanishes and for the high population (40%) of turn at K6–K10, the population of β-strand at residues F2-A11 is between 0.2 and 0.8.Besides the inter-peptide β-sheet structure, sometimes the intra-peptide β-sheet structure appears during the simulations.

## 3. Method

**Figure 5:**
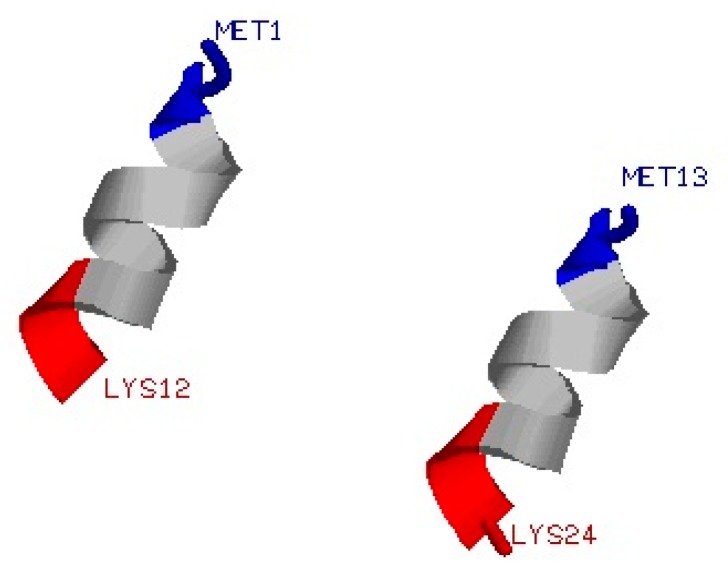
The initial structure of (α-syn12)_2_ used in the T-REMD simulation.

The initial structure of the α-syn12 monomer with a sequence of MDVFMKGLSKAK(residues 1–12 of the human α-synuclein protein)was selected from the NMR-determined micelle bound structure at neutral pH (PDB ID: 1XQ8). A parallel dimer (Figure 5) was constructed by genconf in a GROMACS [30] software package. Molecular dynamics (MD) simulation in the isothermal-isobaric (NPT) ensemble was performed using the GROMACS4.5.3 software package and the GROMOS 43A1 force field [31] with the single point charge (SPC) [32] water model was considered herein. The dimer was dissolved in a rectangular box with the minimum solute-box boundary distance being set to 1.0 nm. Periodic boundary conditions (PBC) were used. The simulations system contained 4775 water molecules. The long-range electrostatic interaction was treated with the Particle-Mesh Ewald (PME) method with a grid spacing of 0.12 and a fourth order interpolation [33,34]. The distance for the coulomb cut-off is 0.9 nm. The van der Waals (VDW) interactions were calculated using a cutoff of 0.9 nm. Protonation states of ionized groups were chosen for physiological pH and acidic pH. At physiological pH, six lysine residue side chains are protonated and two aspartate residues side chains are deprotonated, and four negative counterions (Cl^−^) were added to produce a neutral simulation system. But at acidic pH, both six lysine residue side chains and two aspartate residue side chains are protonated, and six negative counterions (Cl^−^) were added to produce a neutral simulation system. The pressure (set as 1 atm) of the system was kept constant by using a weak coupling algorithm and coupling time of 0.5 ps and isothermal compressibility [35] of 4.575 × 10^−4^ (kJ·mol^−1^·nm^−3^)^−1^. The temperature of the system was kept constant by using velocity rescaling with a stochastic term [36] and using a temperature coupling time of 0.1 ps. The time step for the MD integrator was set to 2 fs and LINCS [37] was applied to constrain all bond lengths. The T-REMD has 24 replicas, which have been simulated at temperatures (in K) from 293 to 358 [38]. The ratios of successful exchange attempts were between 15% and 30% in these simulations. Each replica had been equilibrated at its respective temperature for 100 ps. Then 500 ns T-REMD simulations were performed, with replica exchanges attempted every 2 ps according to the Metropolis criterion. Coordinates and energies have been recorded every 2 ps.

## 4. Conclusions

For α-synuclein protein, the N-terminal residues 1–12 are involved in the dimerization and play a key role in the binding with the lipid membrane. Low pH has been shown to accelerate the aggregation with experimental method. It is important to understand why the α-syn12 peptide dimerization is faster at low pH. To understand the effect of pH on the structural properties of the α-syn12 peptide dimer, we performed 500 ns all atom T-REMD simulations with explicit water molecules at different pH.

From the simulation data, we find that the dimer tends to form the anti-parallel β-sheet conformations rather than the parallel β-sheet at both pH values. As a intrinsically disorder region, the free energy surface obtained from PCA of inter-chain side-chain inverse distances contains six highly populated regions which are nearly flat with a very small barrier at physiological pH, which indicate that these states are widely varied in structure and can easily transit between themselves. However, for the simulation at acidic pH, the free energy surface only contains three highly populated regions. The change of the net charge of Asp2 modulates the conformational ensembles of the dimer. The dimer displays a much more diverse set of structures at physiological pH than at acidic pH. The dimer undergoes a disorder to order transition. The meta-stability of these states cannot be compared directly with the experiment. We also chose other parameters as the reaction coordinates to construct the free energy surfaces. Yet it is difficult to discriminate the anti-parallel β-sheet with different inter-backbone hydrogen. The method developed by Nguyen *et al.* [26] is efficient in the characterization and classification of the conformations.

The contact of intra-peptide Ser9–Lys6 is the major determinant in initiating the transition process. The Lys6–Asp2 contact may prevent the dimerization. Moreover, the respective probabilities for the α-syn12 dimer fall within different regions at different pH and the simulation time, suggesting a faster α to β transition process at acidic pH. The α-syn12 dimer at acidic pH involves more hydrophobic interactions and binds preferentially with the lipid membrane. Furthermore, the anti-parallel states have inter-peptide backbone hydrogen bonds with similar registers as intra-peptide backbone hydrogen bonds found in the representative structure of the isolated monomer. The structure of the monomer has a strong influence on the structure of dimer, which is consistent with the work by Anand *et al.* [39] and Smith *et al.* [17].

It is worth to mention that the dimeric structures of the N-terminal 1–12 residues of the α-synuclein protein may not be relevant to the behavior of the entire α-synuclein. An interesting next step will be a molecular dynamics simulation of the α-synuclein dimer with different membrane.

## Supplementary Materials

Supplementary materials can be found at http://www.mdpi.com/1422-0067/16/07/14291/s1.
