# Supplementary material for: Effect of pH on the Aggregation of α-syn12 Dimer in Explicit Water by Replica-Exchange Molecular Dynamics Simulation
Source: Int J Mol Sci. 2015 Jun 24;16(7):14291–304. doi: 10.3390/ijms160714291 (PMC4519842; doi:10.3390/ijms160714291)
Supplement: Supplementary file 1 [file ijms-16-14291-s001.pdf]

Supplementary Information

1. Effect of pH on the Inter-Molecular States of (α-syn12)$_2$

1.1. Free Energy Surfaces for Different States

Computation of the intra-peptide HB contact probability maps (Figure S1). Inter-peptide HB contact probability maps (Figure S2). States A, B, F, A' and B' are inclined to form extended inter-peptide β-sheet, which is difficult to form intra-peptide HB. State C is composed largely of intra-peptide Lys10–Val3. State D is composed largely of intra-peptide Lys6–Asp2 contact. State E is composed largely of intra-peptide Ser9–Lys6 contact. State C' is composed largely of intra-peptide Lys10–Met5 and Ser9–Lys6 contacts. An interesting note is that the Lys6–Asp2 contact has been characterized in forming the meta-stable β-hairpin associated with the monomer peptide at physiological pH.

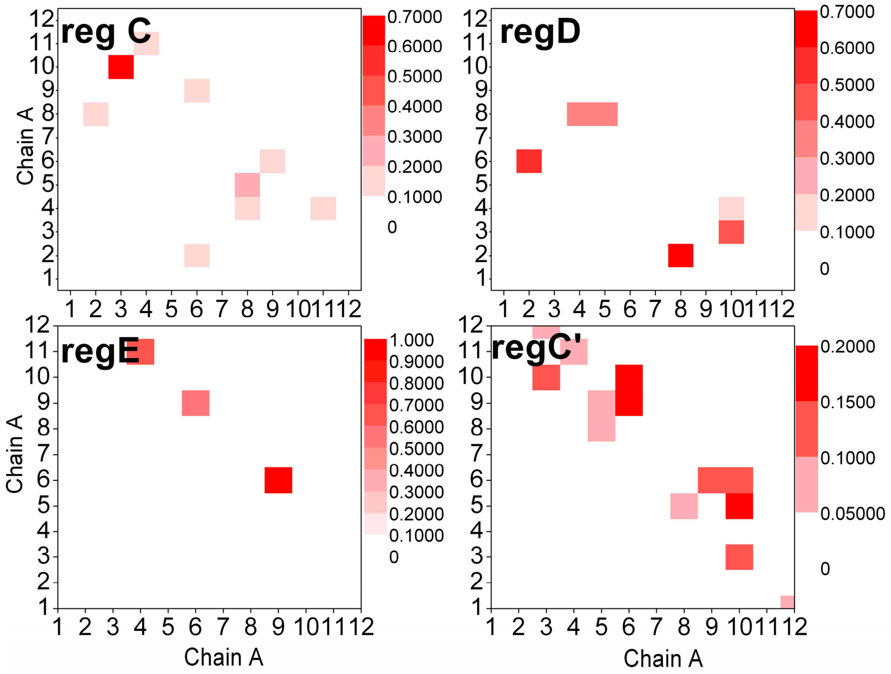

Figure S1. The intra-peptide hydrogen bond contact probability maps for C, D, E and C' regions of the (α-syn12)$_2$ dimer.

Computation of the inter-peptide HB contact probability maps (Figure S2). For the inter-peptide contacts, states A is composed largely of Ser9–Lys6 and Phe4–Ala11 contacts; States Bis composed largely of Lys6–Lys6 and Phe4–Leu8 contacts; while states Fis made up of Asp2–Asp2, Asp2–Phe4, Phe4–Phe4 and Lys6–Leu8 contacts. State A' is composed largely of Ala11–Asp2, Ser9–Phe4, Met5–Leu8 and Val3–Lys10 contacts; State B' is composed largely of Lys10–Asp2, Leu8–Phe4 and Lys6–Lys6 contacts; while state C' is made up of Val3–Asp2, Met5–Phe4 and Met5–Lys10 contacts. At the both pH, hydrophobic residues (such as: Val3, Phe4, Met5, Leu8 and Ala11) involve in the formation of intra-peptide and inter-peptide hydrogen bonds.

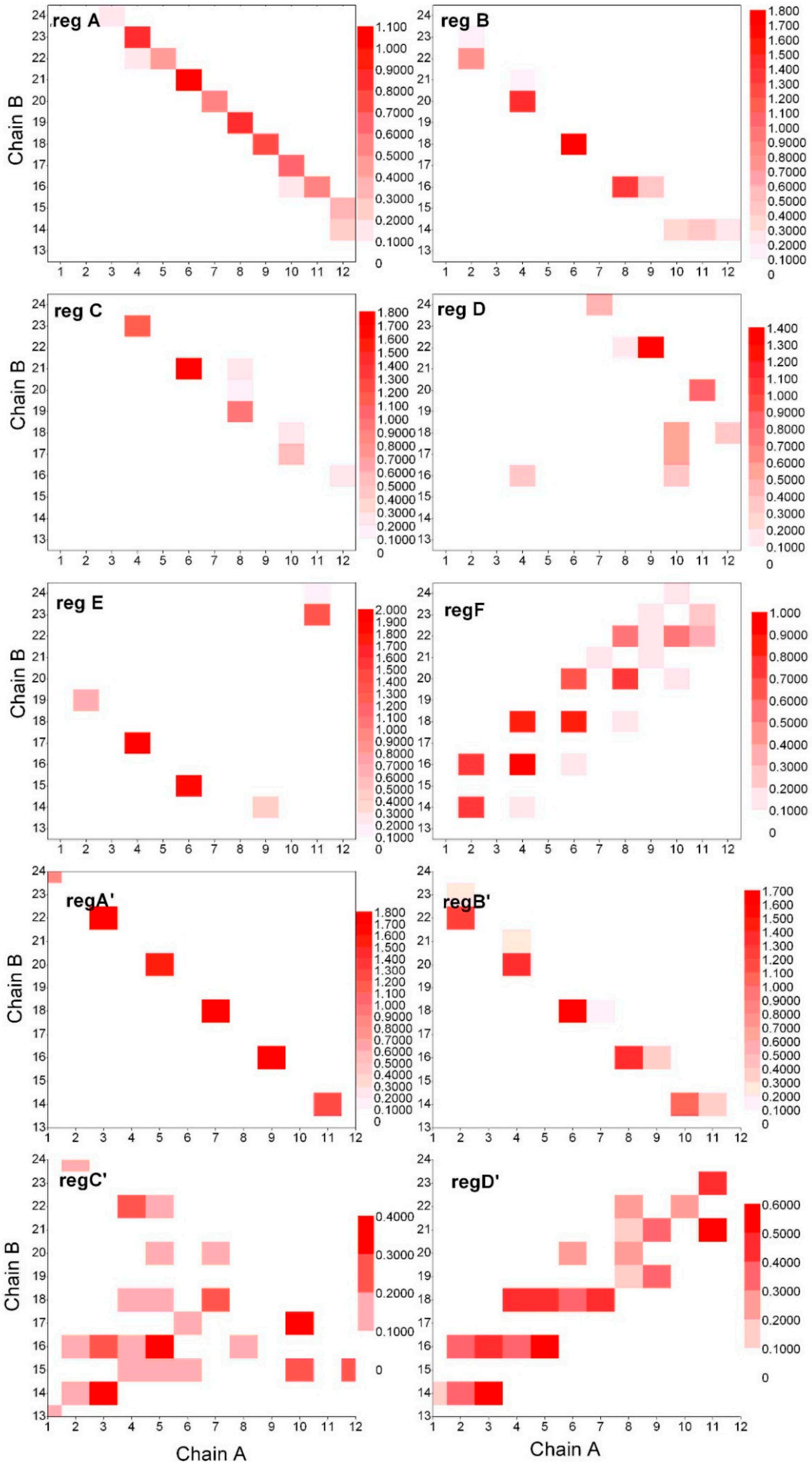

Figure S2. The inter-peptide hydrogen bond contact probability maps for A–F and A'–D' regions of the (α-syn12)$_2$ dimer.

1.2. Possible Transformation Pathways

The intra-peptide HB contact probability maps (Figures S3 and S4) and inter-peptide HB contact probability maps (Figures S5 and S6) for the first 200 ns are analyzed to characterize the conformation transition of dimer. We found that the transition is always initiated by the contact of intra-peptide Ser9–Lys6 at both pH. At physiological pH, Asp2 is negatively charged, which experiences electrostatic attraction from Lys6. For the first 140 ns, the intra-peptide HB contact probability of Lys6–Asp2 is always larger than Ser9–Lys6. However, Asp2 is uncharged at acidic pH, the intra-peptide HB contact probability of Ser9–Lys6, Met5–Leu8 and Val3–Lys10 are relatively higher than others after 40 ns. The α-helix to β-sheet conformational transition at acidic pH is faster than at physiology pH. The Lys6–Asp2 contact may prevent the dimerization.

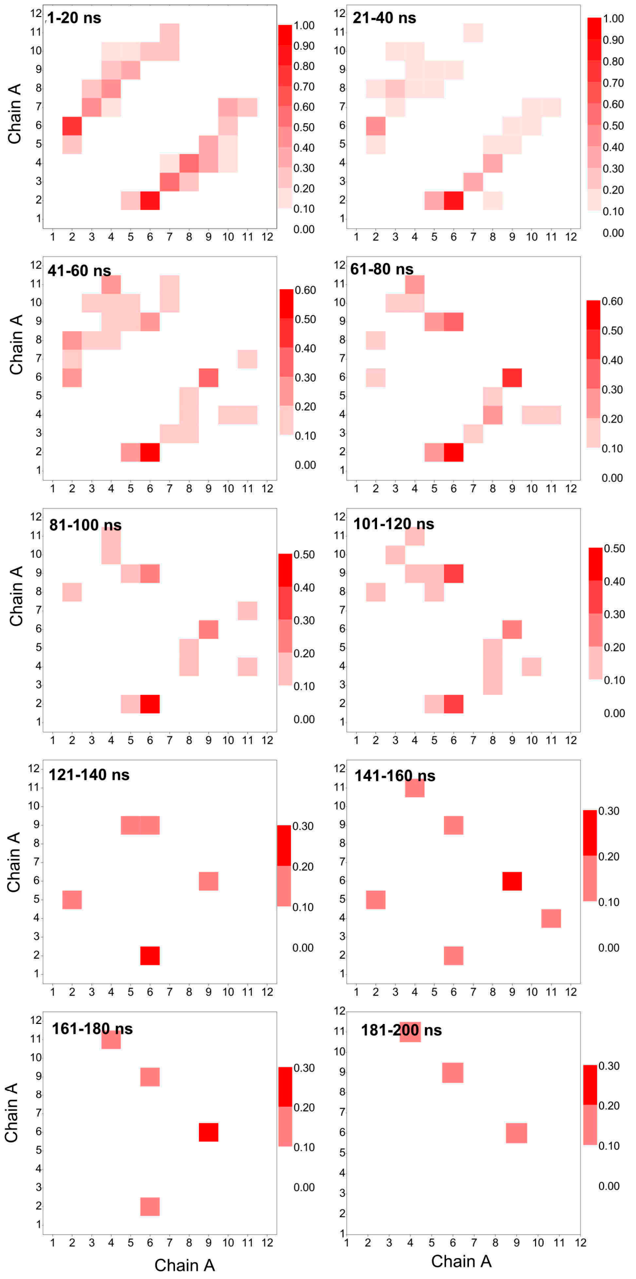

Figure S3. The intra-peptide hydrogen bond contact probability maps of the (α-syn12)$_2$ dimer at the physiological pH for the first 200 ns.

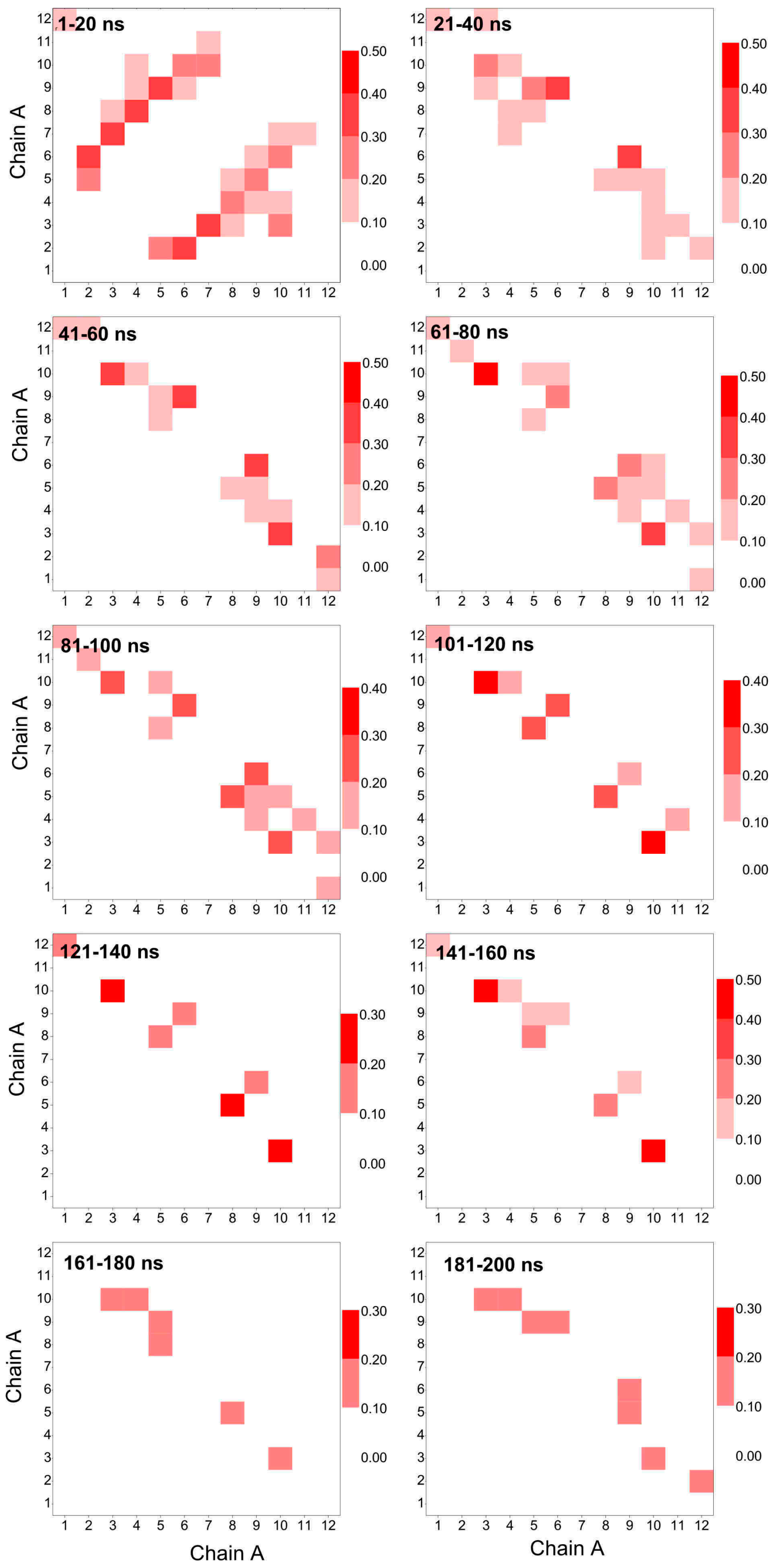

Figure S4. The intra-peptide hydrogen bond contact probability maps of the $(\alpha\text{-syn12})_2$ dimer at acidic pH for the first 200 ns.

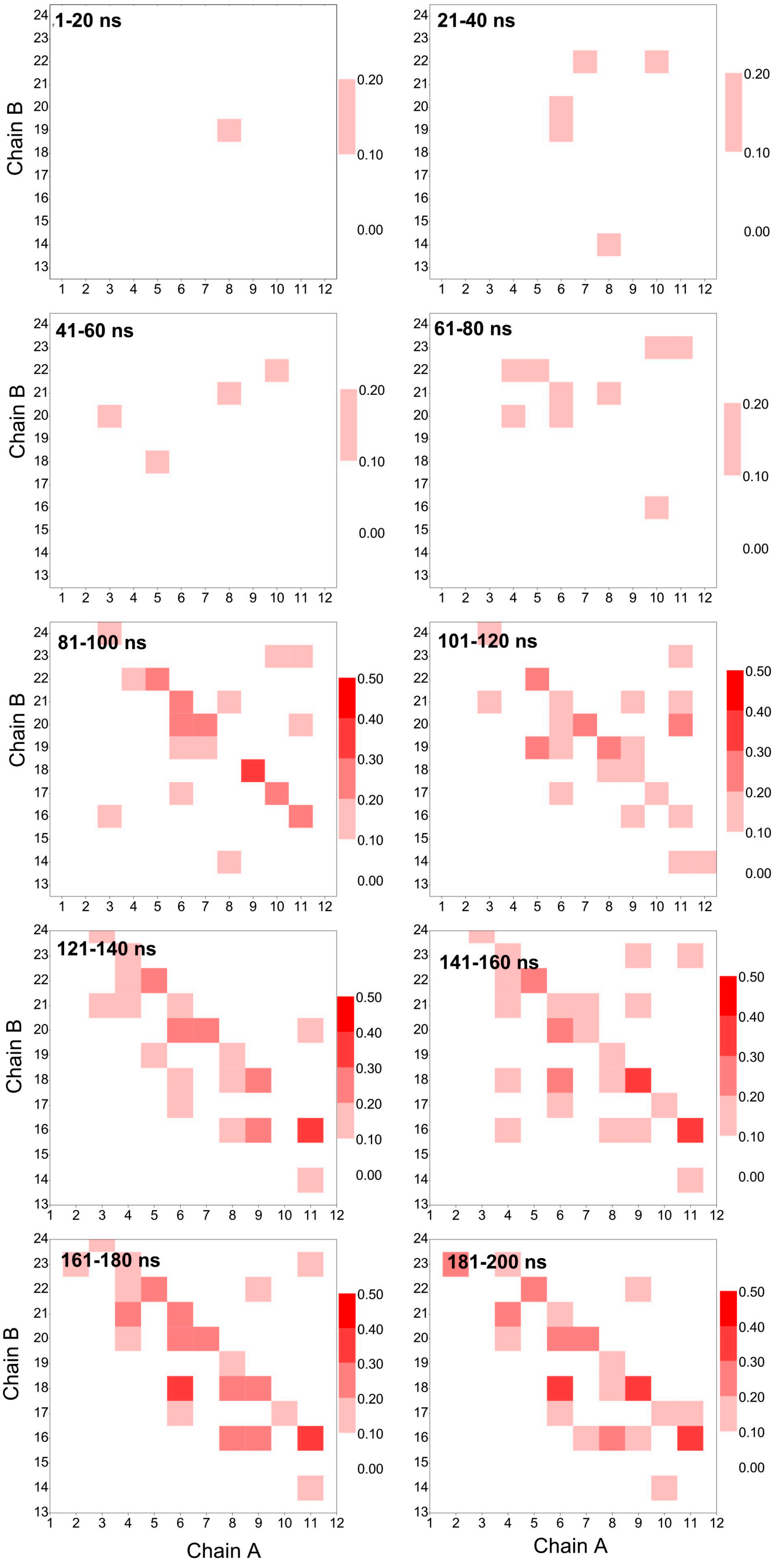

Figure S5. The inter-peptide hydrogen bond contact probability maps of the (α-syn12)$_2$ dimer at the physiological pH for the first 200 ns.

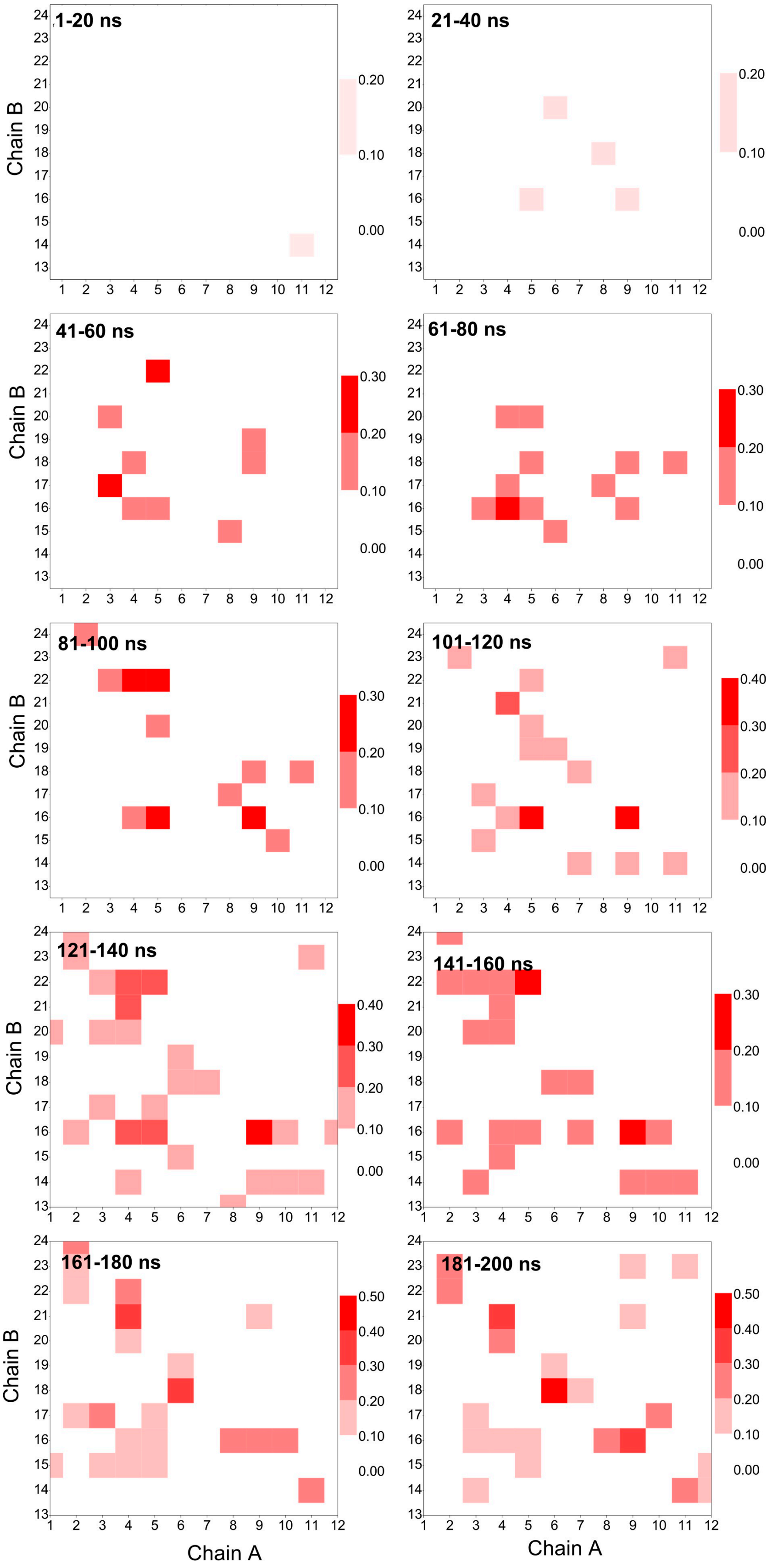

Figure S6. Inter-peptide hydrogen bond contact probability maps of the (α-syn12)$_2$ dimer at acidic pH for the first 200 ns.

2. Intra-Molecular States of the α-syn12 Dimer

We analyzed the residue specific secondary structure propensity, backbone dihedral angle distribution and free energy surface obtained from dPCA, which specify the intra-molecular structures of the dimer.

2.1. Residue Specific Secondary Structure Propensity

The residue specific secondary structure propensity of α-syn12 peptide were calculated with the program STRIDE [40]. The program STRIDE is divided into seven different types of secondary structure: α-helix, 3_{10} helix, π helix, extended conformation, isolated bridge, turn and random coil, in whichα-helix, 3_{10} helix and π helix are referred to be super class Helix while extended conformation and isolated bridge are named as super class Strand. The total 500 ns trajectory set of 300 K were evenly divided into five 100 ns blocks. Finally, the results of our analysis for all 12 residues are shown in Figure S7 (at physiological pH) and Figure S8 (at acidic pH). The distributions sampled by the last three blocks were in general quite similar to each other, which indicated that the last 300 ns was convergence. So at this time, we used the last 300 ns of the trajectory at 300 K to study the structural character of α-syn12 dimer at different pH on one hand. On the other hand, we used the all 500 ns of the trajectory at 300 K to study the transformation pathways of α-syn12 dimer.

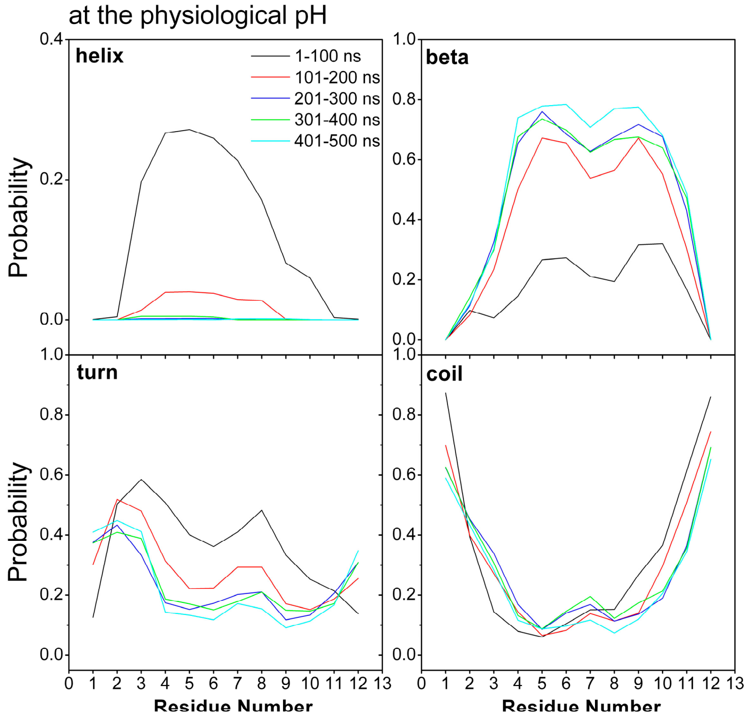

Figure S7. Residue-specific secondary structure propensity of the α-syn12 dimer at the physiological pH.

For α-syn12 dimer at physiological pH, the population of helix is vanishing and a high population (larger than 60%) of β-strand is observed for residues F4–A11. The change of dimer displays a significantly increased β-content compared with monomer at residues F4–A11, especially at residues G6–L7. However, the population of the turn structure centered at residues 2–5 (40%) is consistent with monomer peptide. The monomer peptide [25], one highly populated β-strand structure centered at

residuesF4–K6 and S9–A11 and two highly populated turn structures centered at residues 2–5 and 6–9 were observed. For α-syn12 dimer at acidic pH, the population of helix is vanishing and a high population (40%) of turn at K6–K10, the population of β-strand at residues F2–A11is between 0.2 and 0.8. The turn structure centered at residues 5–8 is consistent with the monomer peptide. The monomer peptide at acidic pH [25], one highly populated β-strand structure centered at residues 2–5 and 8–11 and one highly populated turn structure centered at residues 5–8 were observed. Besides the inter peptide β-sheet structure, sometimes the intra peptide β-sheet structure appears during the simulations.

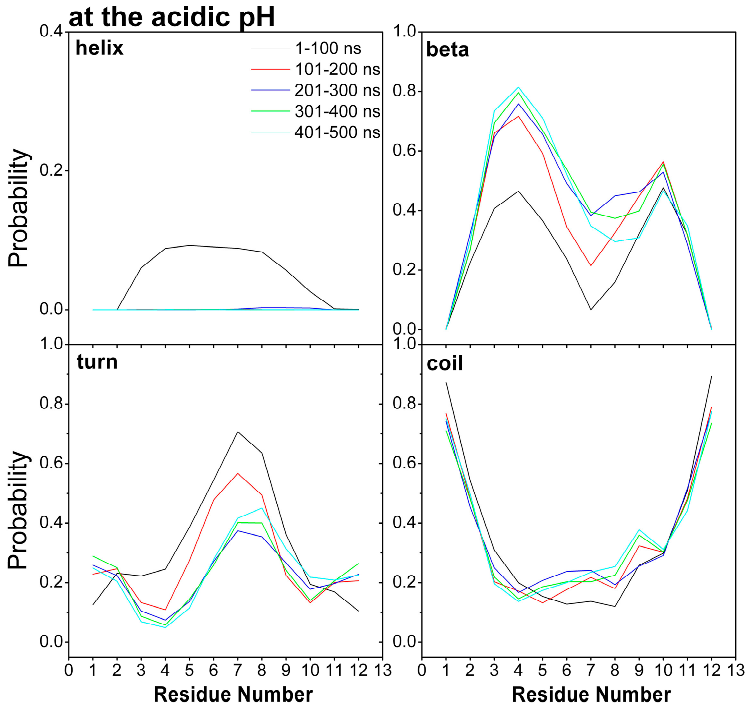

Figure S8. Residue-specific secondary structure propensity of the α-syn12 dimer at the acidic pH.

2.2. The Backbone Dihedral Angle Distributions

Each configuration of the dimer consists of 24 pairs of the backbone dihedral angles. These data for each configuration were pooled together. The distributions of the dihedral (φ, ψ) angles were collected and the potentials of mean force were computed (see Figure S9). Different regions are defined as in the reference [41], α region: −180° < φ < 0° and −120° < ψ < 30°; β region: −180° < φ < 0°, and 90° < ψ < 180° or −180° < ψ < −120°. The probabilities of the (φ, ψ) angles to fall into the α region and the β region were 12% and 70% at the both pH. Our early study indicated that the probabilities of (φ, ψ) angles to fall into the α region and β region were 20% and 58% for the monomer α-syn12. The dimer shows a little bias towards the β sheet structure.

The promoting effect of the dimer can also be derived from the relative depths of the β and α_R minima. Though the β region is always the lowest minimum, the dimer can enlarge the free energy gap between the β region and the α region. For the monomer α-syn12, the free energy gap between the β region and the α region was 2.5 kJ·mol^{-1}. However, for the α-syn12 dimer, the free energy gap was 4.6 kJ·mol^{-1} at the physiological pH and 4.2 kJ·mol^{-1} at the acidic pH.

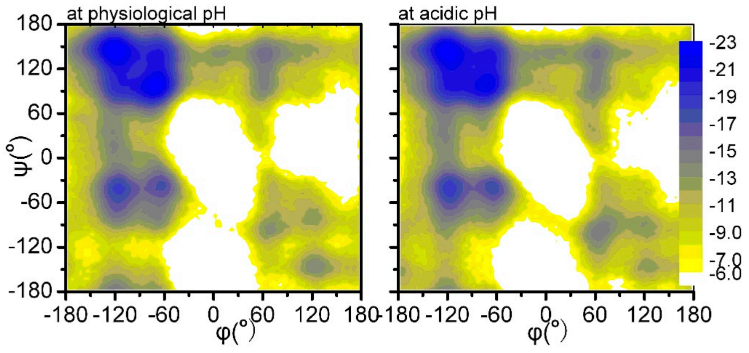

Figure S9. Potential of mean forces along Ramachandran (φ, ψ) angles distributions for the residues at different pH. Neighboring contour lines are separated by 2 kJ·mol^{-1}.

2.3. Free Energy Surface Obtained from dPCA

The backbone dihedral angles can provide important information of local conformation for amino acids. Figure S9 shows the distributions of all backbone dihedral angles. However, it is difficult to identify the representative structure of monomer peptide based on the probabilities of the backbone (φ, ψ) angles. The representative structures of the monomer peptide can be obtained through the Principal component analysis (PCA) in dihedral space. The free energy surface (FES) was constructed using the first two principle components (PC1 and PC2) as the reaction coordinates (see Figure S10). The programs "g_angle", "g_covar" and "g_anaeig" in the GROMACS package were used in the dihedral principal components analysis (dPCA) [42].

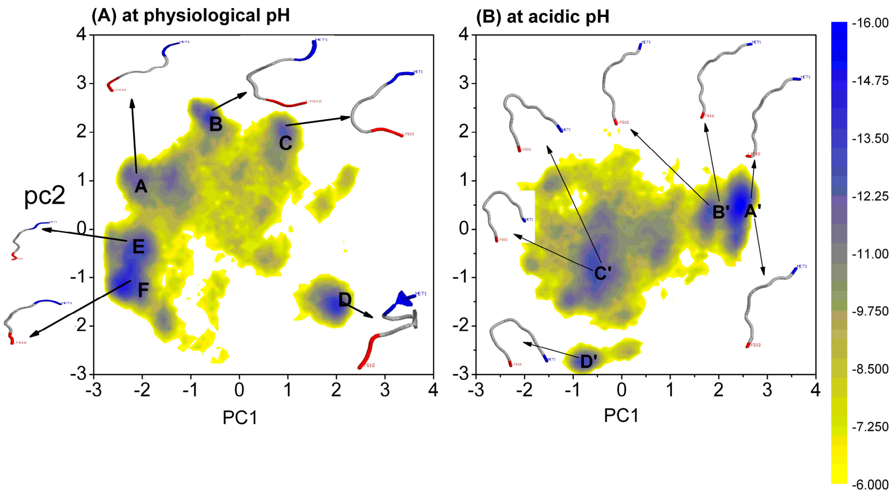

Figure S10. Free energy surface (in kJ/mol) of a single α-syn12 molecule obtained from the simulations of the α-syn12 dimer (**A**) at the physiological pH and (**B**) at the acidic pH projected on the first two principal components PC1 and PC2 obtained from dPCA. The blue part indicates the initial residue.

At the physiological pH, for the last 300 ns of the simulation at 300 K, there were six highly populated regions on the dPCA map centered at (−2.0, 0.9), (−0.6, 2.3), (0.8, 2.0), (1.9, −1.5), (−2.2, −0.8), and (−2.4, −1.1) (cited as A–F in Figure S10A), the corresponding free energy were −12.2, −13.6, −12.7, −14, −14.1 and −14.7 kJ/mol, respectively. At the acidic pH, for the last 300 ns of the simulation at

300 K, there were four highly populated regions on the dPCA map centered at (2.4, 0.4), (1.8, 0.3), (−0.5, −0.6), and (−0.8, −2.8) (cited as A'–D' in Figure S10B), the corresponding free energy were −15.9, −13.1, −13.2 and −13.3 kJ/mol, respectively. Besides, the dPCA map also contains the other basins with slightly high free energy. The relatively flat dPCA map indicated that it is easy for the conversions to occur among the conformers. The corresponding representative structures are shown in Figure S10. These six states are inclined to form extended conformation, which are different from those sampled by the monomer peptide simulated alone because of the inter peptide interactions.
